# A novel structure for online surgical undergraduate teaching during the COVID-19 pandemic

**DOI:** 10.1186/s12909-020-02236-9

**Published:** 2020-09-22

**Authors:** P. C. Chandrasinghe, R. C. Siriwardana, S. K. Kumarage, B.N.L Munasinghe, A. Weerasuriya, S. Tillakaratne, D. Pinto, B. Gunathilake, F. R. Fernando

**Affiliations:** Department of Surgery, Faculty of Medicine, University of Kelaniya, Kelaniya, Sri Lanka

## Abstract

**Background:**

The Covid-19 pandemic necessitated the delivery of online higher education. Online learning is a novel experience for medical education in Sri Lanka. A novel approach to undergraduate surgical learning was taken up in an attempt to improve the interest amongst the students in clinical practice while maximizing the limited contact time.

**Method:**

Online learning activity was designed involving medical students from all stages and multi consultant panel discussions. The discussions were designed to cover each topic from basic sciences to high-level clinical management in an attempt to stimulate the student interest in clinical medicine. Online meeting platform with free to use basic plan and a social media platform were used in combination to communicate with the students. The student feedback was periodically assessed for individual topics as well as for general outcome. Lickert scales and numeric scales were used to acquire student agreement on the desired learning outcomes.

**Results:**

A total of 1047 student responses for 7 questionnaires were analysed. During a 6-week period, 24 surgical topics were discussed with 51 contact hours. Eighty-seven per cent definitely agreed (highest agreement) with the statement ‘students benefitted from the discussions’. Over 95% have either participated for all or most sessions. A majority of the respondents (83.4%) ‘definitely agreed’ that the discussions helped to improve their clinical sense. Of the total respondents, 79.3% definitely agreed that the discussions helped to build an interest in clinical medicine. Around 90% agreed that both exam-oriented and clinical practice-oriented topics were highly important and relevant. Most widely raised concerns were the poor Internet connectivity and limitation of access to the meeting platform.

**Conclusion:**

Online teaching with a novel structure is feasible and effective in a resource-limited setting. Students agree that it could improve clinical interest while meeting the expected learning outcomes.

## Background

The COVID-19 pandemic has posed unprecedented challenges globally. Provision of formal higher education was significantly affected due to the closure of universities as a measure to curtail disease spread. The lockdown affected medical education on several fronts. Students had to be kept away from the hospitals and the teaching staff was overwhelmed with COVID-19 related healthcare provision. Globally, universities moved into adopting online teaching programmes for undergraduate and postgraduate teaching to overcome these difficulties [1, 2]. In a resource-limited setting such as in Sri Lanka, some of the challenges faced were lack of access to the Internet and availability of established institutional online learning platforms. Amidst the COVID19 pandemic, the department of surgery took a novel approach to maximise the resources by arranging teaching sessions applicable to students of all levels (1st-year medical students to postgraduate trainees) using freely available meeting platform Zoom®. Open access social media platform Facebook® with a closed group was set up to communicate with the students more efficiently and to upload the recorded session. Although the main focus of the session was teaching the final year medical students, by opening the sessions to the others, we intended not only to provide formal teaching but also to develop an interest in clinical surgery amongst the students. This study analyses the student acceptance and attitude towards this low-cost resource maximizing approach.

## Method

### Study sample

From the 1st of April 2020 up to 15th of May 2020 the department of surgery conducted 16 teaching sessions on clinical surgery topics. A closed group was created on Facebook® under the title ‘Young surgeons forum’ to establish direct communication with a large group of students. An open invitation was sent to all the students in the university. A total of 754 students (from a total of 800) joined the Facebook® group voluntarily.

### Method of teaching

The lesson plan for each topic was designed to cover a wide range beginning with the basic science components and finally concluding with advanced management. Teaching sessions were held using the Zoom® video conferencing platform which provides basic plans for free of charge. Junior medical students were assigned the task of presenting the basic science explanation on a topic while the senior students (4th and final year) were expected to present a clinical case for discussion. Patient management component was more focused on the senior students and finer operative surgical details part of the discussion was gradually shifted to the postgraduate trainees. Each session lasted for 120 to 180 min. Four different methods were used for teaching;

1. Power-point slides as a guide and maintaining discussion amongst the academic members highlighting the key areas in -between.
2. As a panel discussion on a topic among teachers.
3. Case presentation based discussion.
4. Discussion based on a single -best -answer type questions.

### Contribution from academic staff members

A minimum of 3 to 5 staff members were involved in each session. All sessions were conducted in the form of a discussion at times amongst the teachers themselves and with student participation. After discussing every key area, one member highlighted the required core knowledge areas. Positive and negative feedback was given on the student presentations.

### Evaluation

Periodical feedback forms were posted on the social media platform. Student opinion on relevance, importance and the quality of the teaching activity related to individual topics were assessed. A general questionnaire to assess the acceptance of the programme was also distributed amongst the participants. All responses were recorded anonymously and analyzed at the end of 6 weeks.

## Results

A total of 1047 student responses for 7 questionnaires were analysed. All sessions recorded a participation of 250 to 300 students. Use of the software platform without subscription limited the number of participants to 300, which included staff members and postgraduate trainees of the unit. A total of 24 surgical topics were discussed within a 6-week period with 51 contact hours. A significant majority of the respondents were from the final year (84%; *p* < 0.05).

**Fig. 1:**
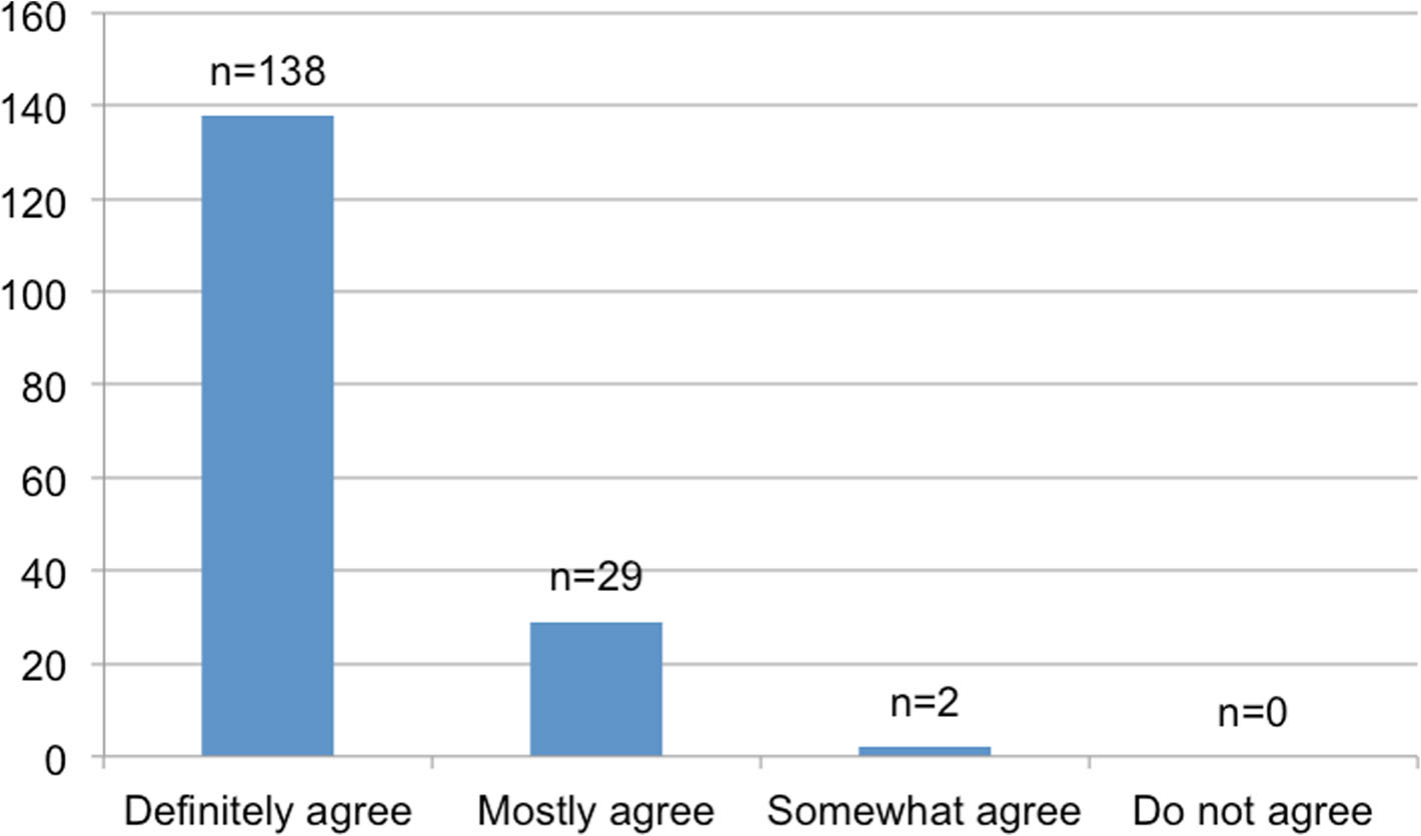
Agreement to the statement, ‘*students benefitted from the discussions*’, in the general questionnaire (*n* = 169)

**Fig. 2:**
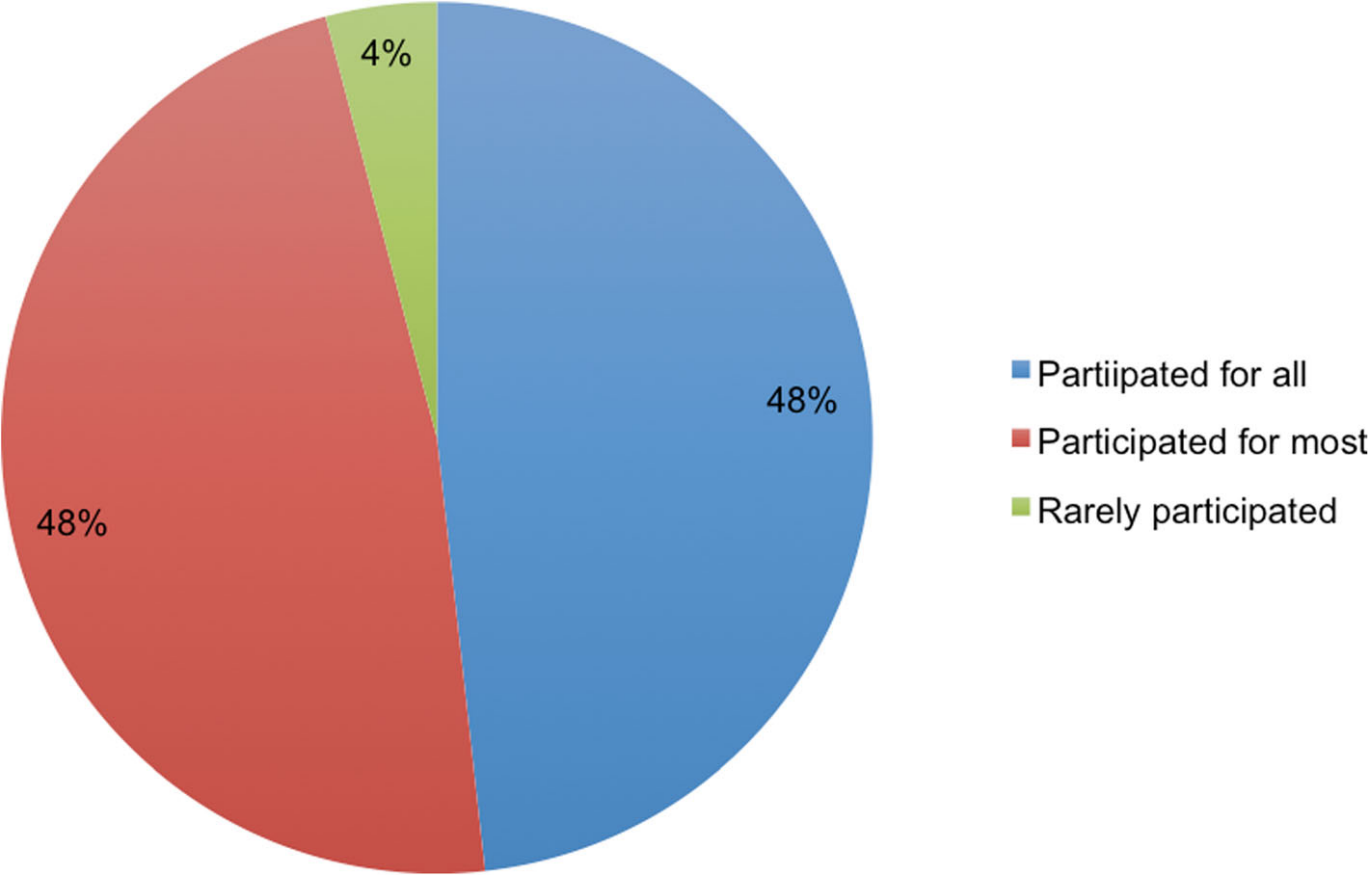
Frequency of participation for online discussions amongst the responders

**Fig. 3:**
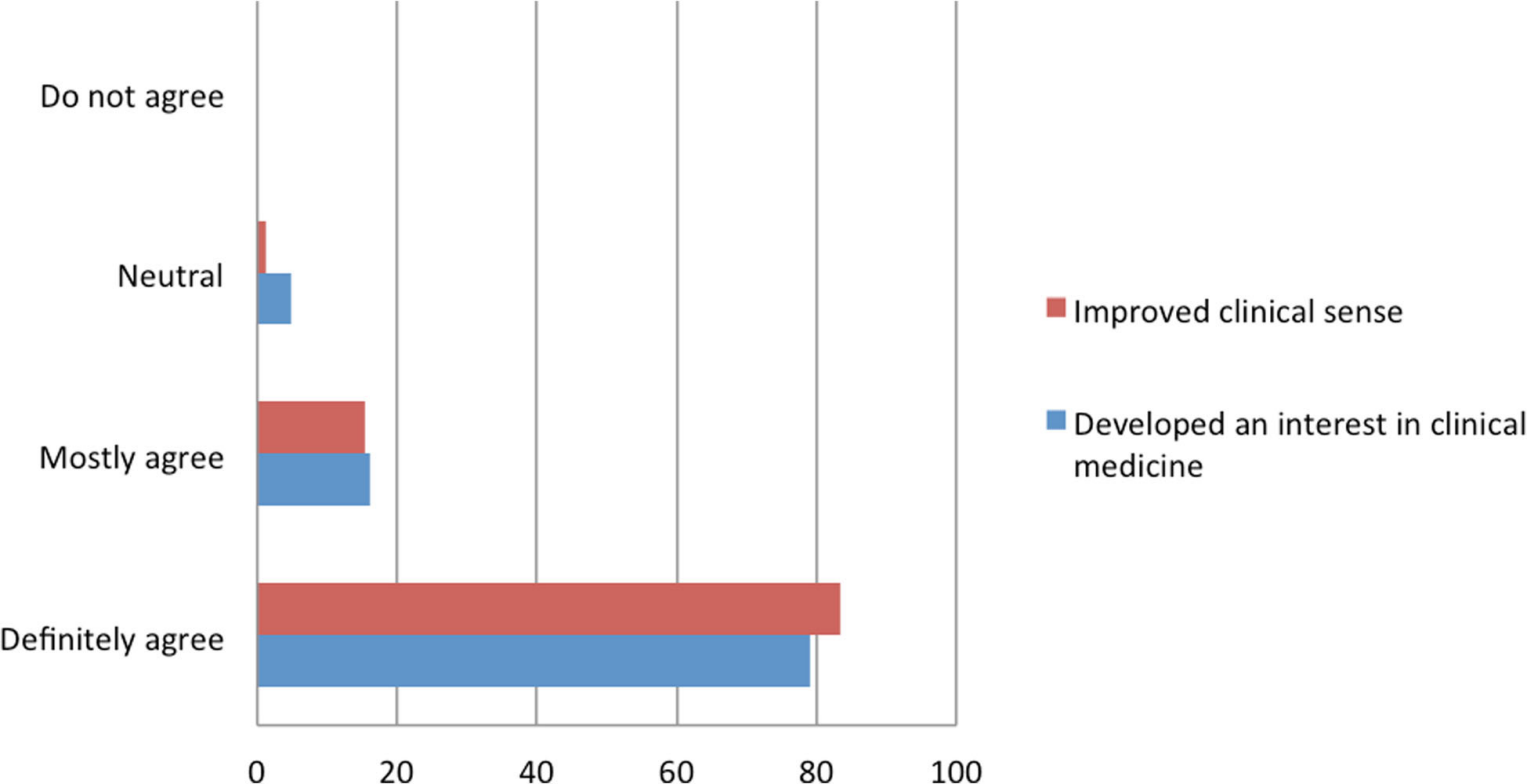
Student agreement (percentage) on improvement in clinical sense and developing an interest in clinical medicine through the novel teaching method

In response to the general assessment questionnaire, 87.1% definitely agreed (highest agreement) with the statement ‘students benefitted from the discussions’ on a Likert scale while another 17.2% mostly agreed with the same (Fig. 1). Forty-eight per cent of the respondents have participated in all sessions while another 47.6% have participated for most (Fig. 2). Over 98% were in agreement with the fact that the discussions improved their clinical sense, with a majority response of (83.4%) ‘definitely agree’ and another 15.4% with a response of ‘mostly agree’ (Fig. 3). All respondents (70.6% - Very relevant; 26.4% - somewhat relevant) agreed that the discussion points were in line with their learning objectives. Of the total respondents, 79.3% definitely agreed with the fact that the discussions helped to build an interest in clinical medicine while a 16% agreed somewhat (Fig. 3). The rest took a neutral stance to this question.

**Fig. 4:**
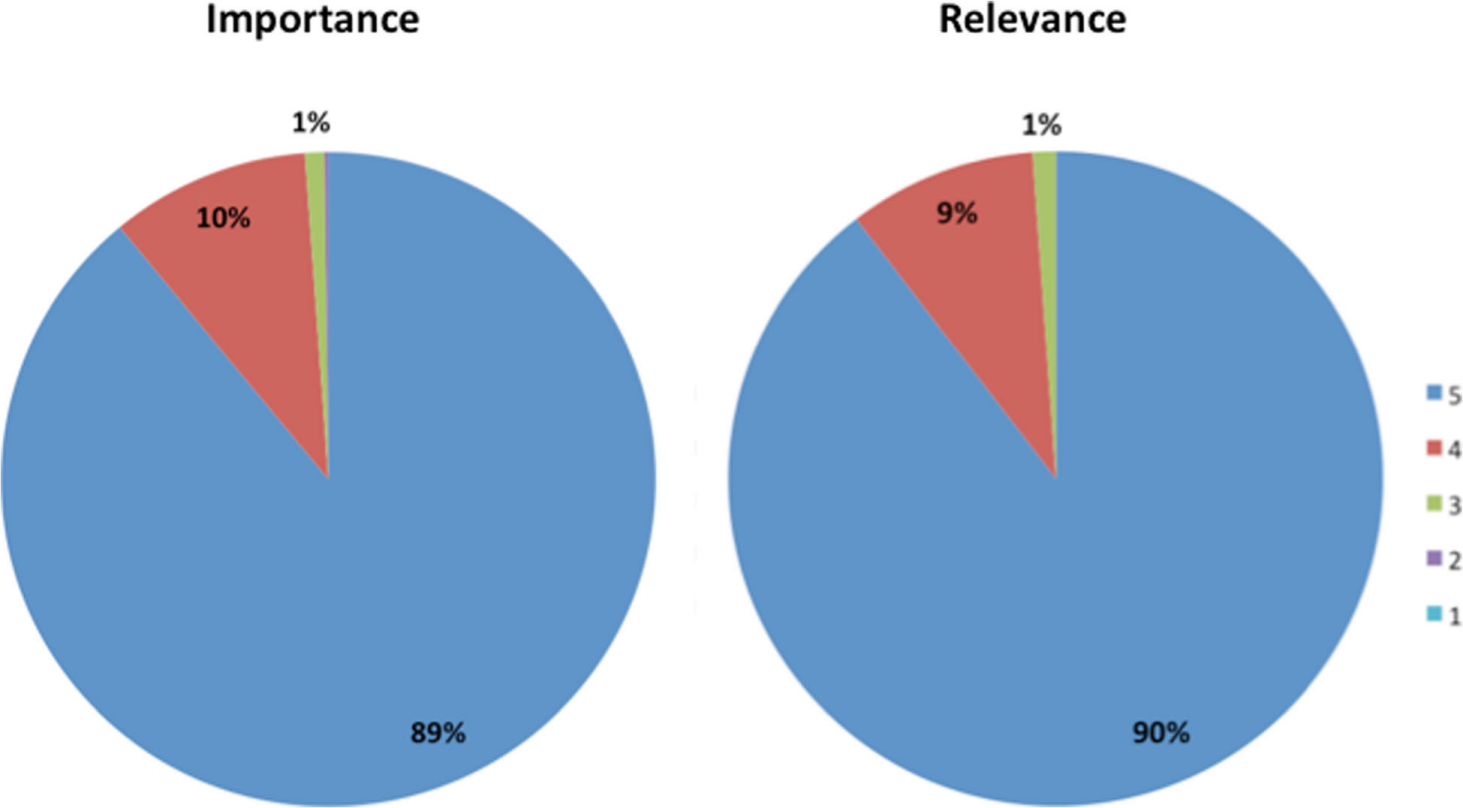
Importance and relevance of the discussion topics to academic work on a numeric scale from 1 to 5 (5- very important, 1- not important)

**Fig. 5:**
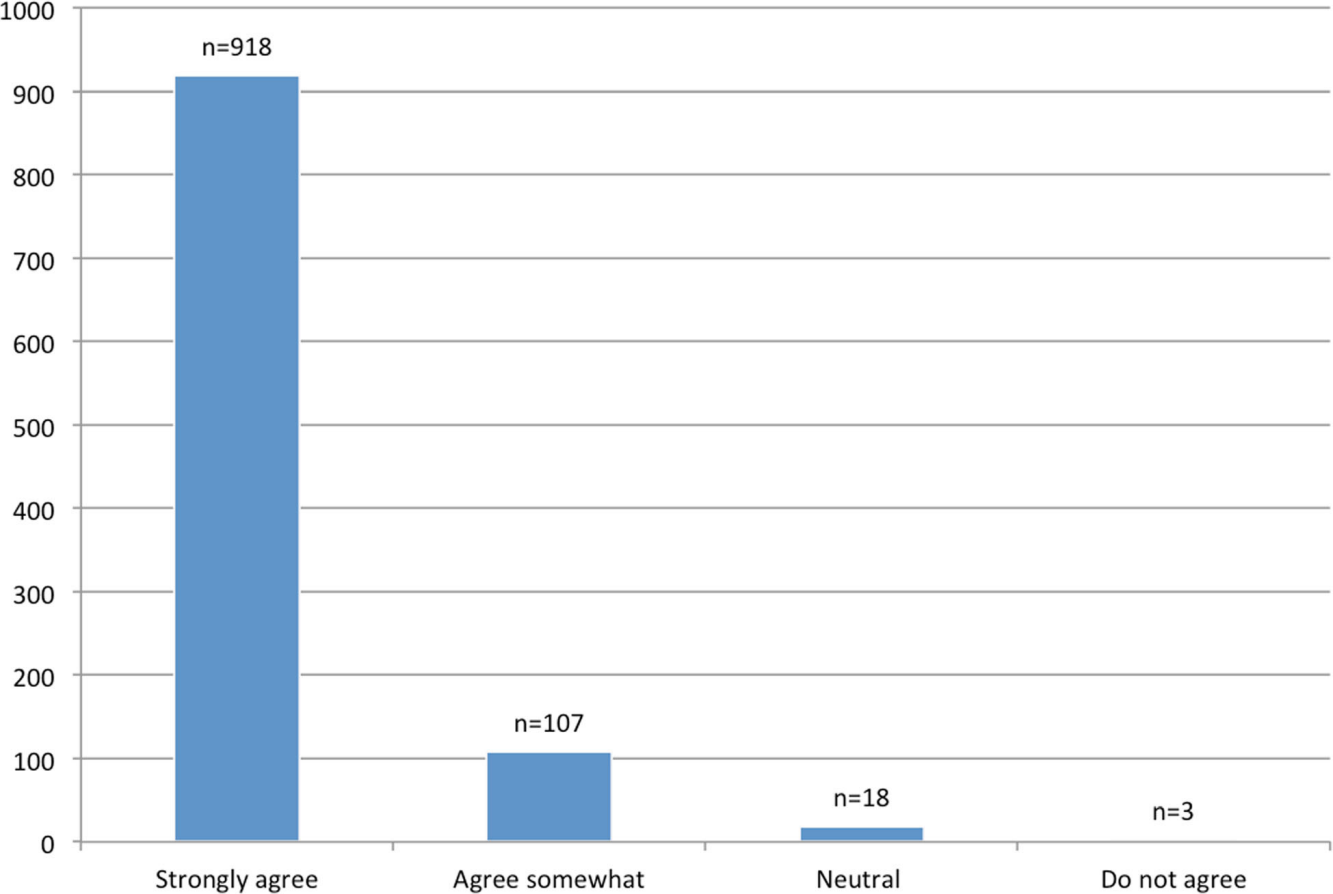
Cumulative agreement of the participants on the statement: ‘*discussions helped to improve understanding and knowledge on the topic’*

In cumulative responses to topic-based feedback, 90% responded with the highest score on the numeric scale (1 to 5) to the relevance of the topics while 89% scored the same for the importance of the topic (Fig. 4). On the same scale, 96% had scored above 4 to the query ‘how well the discussions were conducted’. On a Likert scale, 88% strongly agreed (highest agreement) that the learning activity helped them to gain knowledge and understanding on the topic (Fig. 5). Eighty-two per cent were in strong agreement with the fact that highlighting of important points and summarising the lesson was done adequately. Analyzing the cumulative responses to examination-oriented (model question based) discussions versus more clinically oriented panel discussions revealed a similar level of positive responses (Table 1). Around 90% were of the view that both exam-oriented and clinical practice-oriented topics were highly important and relevant. Most prominent concerns with regards to online learning activities were the Internet connectivity and limitation of access to the online meeting platform. Out of 170 responses for the general assessment questionnaire, 31% complained about not having optimal Internet connectivity and 25% had faced difficulty at some point in time due to the limited number of participants for a session on the meeting platform. None of the participants has mentioned difficulty in using the software platform.

**Table 1:**
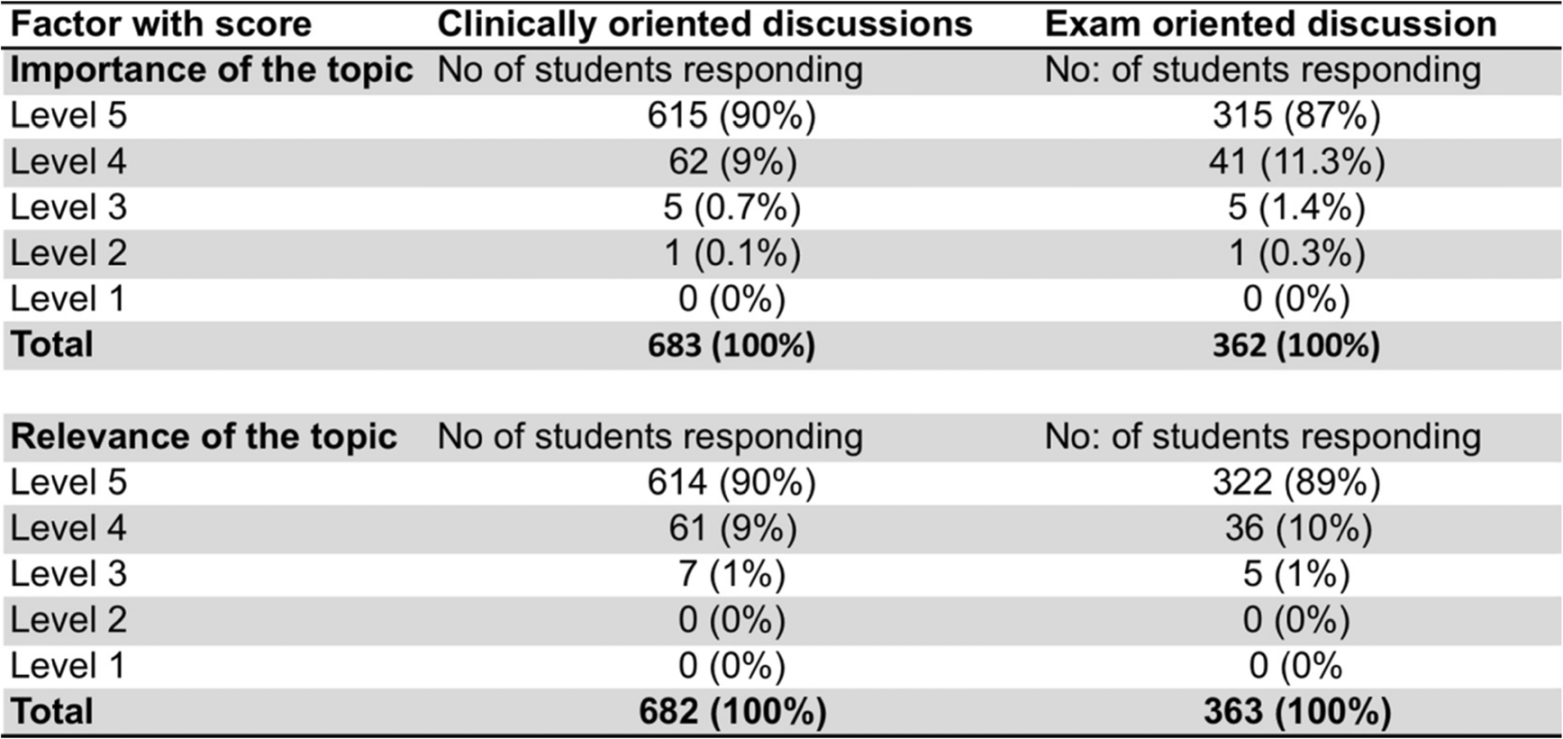
Analysis of the student perception on importance and relevance of the discussion based on their orientation.

## Discussion

Principles of higher education have changed significantly over the last few decades [3]. With the conversion of the traditional classroom to a student-centred learning environment, obtaining student feedback on all educational activity has become integral [4]. Sudden adaptations had to be made globally to the methodology and content in delivering education, to meet the challenges of COVID19 [5, 6]. In the Sri Lankan context, online teaching is a completely new experience for both students and teachers. The novelty of the current approach was that we used a multimodal teaching approach using discussion-based sessions involving an audience with a wide range, focusing from basic sciences to advanced clinical care. As there was no clear date of restarting the formal academic program, students were participating in the sessions without the pressure of exams. Acceptance of the activity by students is a key component in these changes, which made feedback a priority. In the current study, around 90% were in complete agreement that students benefitted from the activity (87.1%), the topics were relevant (90%) and important (89%) to their course. Although around half the respondents have participated in all scheduled sessions, all sessions reached maximum participation on the meeting platform.

A major concern in adopting an online learning activity in the Sri Lankan context is resource limitation. Quality of Internet connectivity was the major issue faced by many participants although not many have complained of lack of access. Most of the students gained access through their mobile phones. Lack of bandwidth and poor processing speed in mobile connections leads to frequent disconnections, poor video quality and poor downloading speeds. This is a frequent setback reported in resource-poor setting [7, 8]. Authors observed that around 25% of the respondents raised the issue of limiting the number of participants to 300 for individual sessions. Access to the meeting platform was provided through the Lanka Education and Research Network (LEARN), which is a government-funded agency. Free access to the Zoom® platform enabling 300 participants and 3-h sessions were provided to all university students and staff by LEARN during the pandemic. This facility enabled us to hold large group discussions which otherwise would have required the purchasing of premium packages. Importantly none of the students has raised a concern regarding difficulties in operating the software. Considering the positive response of students on online teaching, supporting them with a program to purchase high-quality devices will improve their learning capacity.

Use of the social media platform Facebook® to communicate with the students was undertaken to reach a large platform at once. The students and the teachers are more familiar with social media compared to formal computer-assisted learning platforms. Student involvement in the communication was also high due to the user-friendly interface and familiarity of the platform. Use of popular social media in medical education during COVID19 was reported by Sethi et al. and others with great success [1, 2]. These observations will be useful when designing online learning platforms in the future.

Usually, a single teacher carries out undergraduate teaching in medical education. One intention of the novel structure was to make maximum use of the limited contact time with the students. On the other hand, we intended to implement a teaching method that could stimulate the student interest in clinical sciences. Participation of a panel of teachers with active cintributions, provided the students with a new experience. There is a lack of data on the effectiveness of panel discussion on a large group of students in undergraduate medical education [9]. Participation of multiple teachers was practically possible as the teaching took place after working hours when all were free of their clinical commitments.

Exam-oriented learning amongst the students not only increases the stress levels but also go against the primary objective of medical education [10–12]. The issues with the concept of ‘teaching to test’ has been discussed previously [13]. The tradeoff between memorizing assessment oriented facts and learning practice-based skills is an ongoing debate. It is well known that most medical students resort to either proprietary based applications or informal teaching from senior colleagues to score at exams [13–15]. Although ‘peer teaching’ can be effective, it could result in the spread of misinformation when unregulated. The discussions were structured to span the entire clinical spectrum of the disease process to stimulate the students to think outside of the traditional scope. It is encouraging to see around 80% being in complete agreement on the fact that the activity helped to improve both the clinical sense and interest in clinical medicine. The balance between basic science and clinical skills in medical education is another long-discussed topic. The basic science component of medicine is better understood when it is combined with the clinical application [13]. The modern integrated syllabus is aimed at achieving a perfect mix between the two components [16, 17]. However, our personal experience has been that students tend to shift away from clinical exposure based learning and focus more on the exam-oriented aspect. In the current model, deviating from the traditional online lectures accommodated variety and theinvolvement of several lecturers allowed to carryout discussion-based teaching. Question and answer sessions amongst lecturers allowed the highlighting of important aspects in a topic more effectively and efficiently. Effectiveness of the activity is validated by 88% strongly agreeing that the activity improved their understanding of the topic and student comments on the feedback questionnaires (Fig. 5). Also in this cohort of medical graduates, a similar response was received for examination-oriented and clinical practice-oriented discussions, indicating that the balance can be achieved through a structured learning programme.

## Conclusion

A novel structure was adopted to conduct online learning activity for medical students during the COVID-19 pandemic. The objective was to improve the interest in clinical medicine by improving the clinical sense while continuing learning activities during the lockdown. The programme was structured to involve a wider audience, multiple teachers and the breadth of discussions spanning from basic sciences to advanced clinical management. Student acceptance of the novel structure was highly satisfactory. Feedback data indicated that the learning sessions were able to achieve the learning objectives while improving the clinical sense and the interest in clinical medicine amongst the students.

## Data Availability

The data is available at 10.6084/m9.figshare.12609023.v1

## Abbreviations

COVID19: Coronavirus disease 2019
LEARN: Lanka Education and Research Network
